# The complete chloroplast genome sequence of *Vernonia amygdalina* Delile

**DOI:** 10.1080/23802359.2021.1902411

**Published:** 2021-03-19

**Authors:** Fang Zhou, Keke Lan, Xiangrong Li, Yu Mei, Shike Cai, Jihua Wang

**Affiliations:** a Key Laboratory of Crops Genetic Improvement of Guangdong, Crops Research Institute, Guangdong Academy of Agricultural Sciences, Guangzhou, China; b Guangdong Academy of Agricultural Sciences, Guangzhou, China; c Agricultural and Rural Bureau of Luhe County, Shanwei, China

**Keywords:** *Vernonia amygdalina* Delile, chloroplast genome, medical plant

## Abstract

*Vernonia amygdalina* Delile is widely used in folkloric medicine for the treatment of various diseases. In this study, the complete chloroplast genome of *V. amygdalina* Delile was reported, which was assembled and annotated base on genome high-throughput sequencing data. This work provided the clues for the taxonomy of the herb and the potential to utilize the chloroplast genome sequence as a new study target. The length of *V. amygdalina* Delile chloroplast genome was 153,133bp, with two single-copy regions, each has the length of 84,245bp and 13,152bp respectively. This region were separated by two inverted repeat regions with 27,868bp in length. It was predicted to consist of 131 genes in total, including 86 protein-coding genes, 37 tRNA genes, 8 rRNA genes with GC content at 37.68%. Phylogenetic analysis by RAxML (Random Axelerated Maximum Likelikhood) showed *V. amygdalina* Delile is closest to *Sonchus webbii* in *Compositae*.

*Vernonia amygdalina* Delile is commonly known as the South African leaf,which is native to tropical Africa. It was transplanted to China and distributed in Guangdong, Fujian,Hainan and other places. *V. amygdalina* Delile contains a variety of active ingredients, such as saponins, flavonoids, phenolic acids, steroids, alkaloids, coumarins, lignans, xanthones, anthraquinones, terpenes and sesquiterpenes etc as the traditional herb and folkloric medicine used widely (Alara et al. 2018; Madzuki et al. 2019; Ifedibaluchukkwu et al. 2020). However, current research on *V. amygdalina* Delile is insufficient, to detect the information, including genetic background, will greatly promote the precise application of the herb and accumulate the fundamental knowledge of this valuable herb. In this study, we assembled the complete chloroplast genome of *V. amygdalina* Delile local plant to provide genetic sources for further research.

The leaves of *V. amygdalina* Delile were collected from the herb garden in Guangdong Academy of Agricultural Sciences (Guangzhou, China, N23.1459, E113.3498). The young leaves were collected and frozen by liquid nitrogen quickly, then stored in −80 °C refrigerator. We extracted and strored the genomic DNA in Key Laboratory for Crops Genetic Improvement of Guangdong in Guangdong Academy of Agricultural Sciences(specimen code Nfy 2019) by plant genomic DNA kit (Omega) and sequenced by the Novaseq platform (Illumina, San Diego, CA) following the user manual. The sequence was assembled by GetOrganelle (Jin et al. 2019) and annotated by Geseq (Tillich et al. 2017). After that, the data were submitted to GenBank and assigned the accession number MT795180.

The chloroplast genome of *V. amygdalina* Delile was found to possess a total length 153,133bp, with the GC content of 37.68%, including 86 gene coding regions, 37 transfer RNA(tRNA) coding regions and 8 ribosomal (rRNA) coding regions. The chloroplast genome composed of 84,245 bp of large single copy (LSC) region, 13,152 bp of small single copy (SSC) region, and 27,868 bp of a pair of inverted repeated (IR) regions.

To compare the relationship between *V. amygdalina* Delile and other 18 genera in *Compositae* order, the sequence data obtained from NCBI GenBank, and been analyzed by MAFFT(Multiple Alignment using Fast Fourier Transform) program(Katoh and Standley 2013).The phylogenetic tree was constructed with the full chloroplast sequence of these species by RAxML (Random Axelerated Maximum Likelikhood) (Figure 1)（Stamatakis, 2014）.The results showed that *V. amygdalina* Delile was closest to *Sonchus webbii* (NC-042383). The complete chloroplast genome sequence of *V. amygdalina* Delile will provide a genomic resource for the conservation genetics of this species and wide the development of its medicinal utilization research.

**Figure 1.**
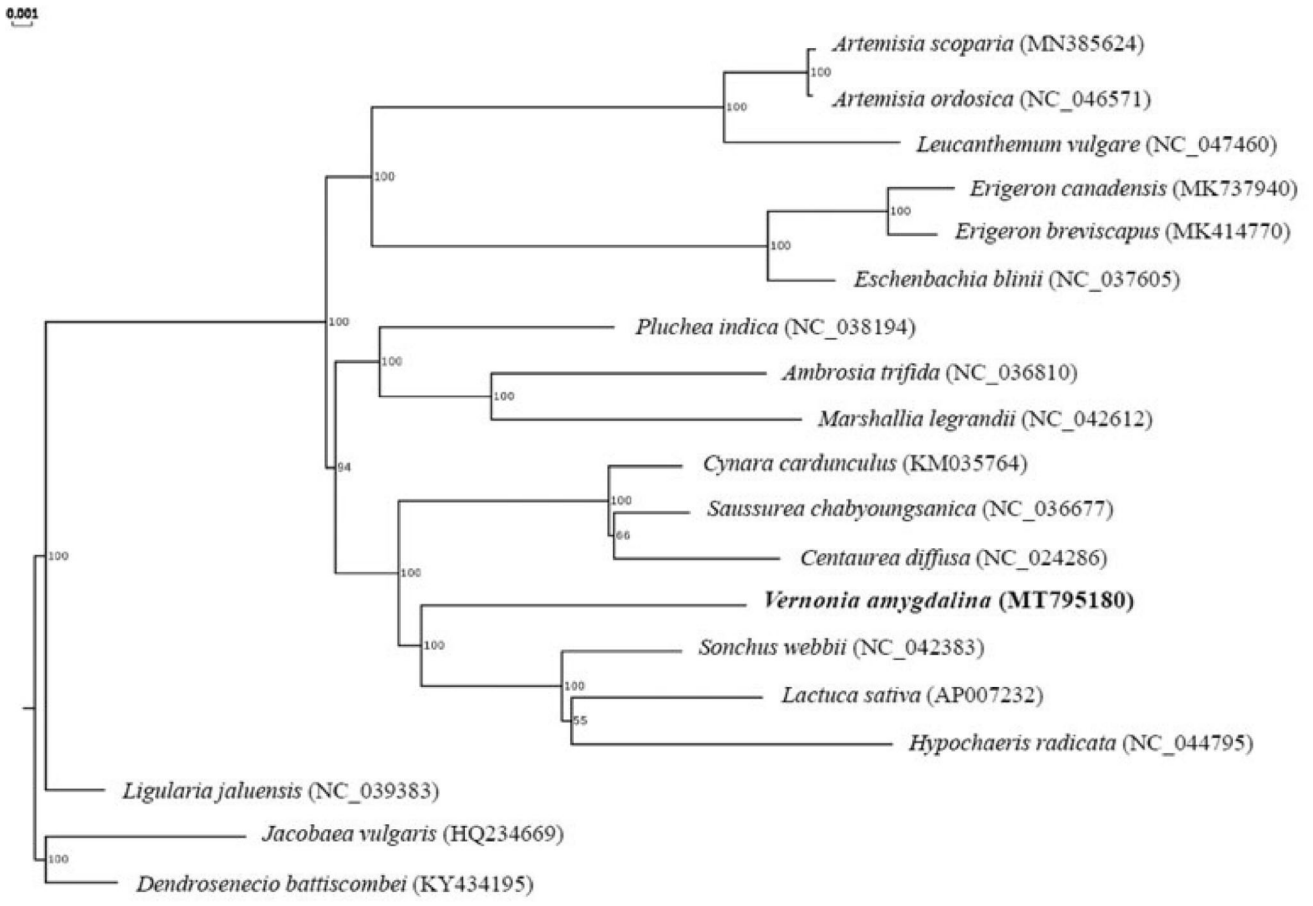
The phylogenetic tree of *V. amygdalina* Delile with other species based on the complete chloroplast sequence. Numbers above each node were bootstrap values.

## Data Availability

The genome sequence data that support the findings of this study are openly available in GenBank of NCBI at (https://www.ncbi.nlm.nih.gov/) under the accession no. MT795180. The associated SRA numbers are SAMN17221994.
